# Cellulose Modified with Polyethylenimine (PEI) Using Microwave Methodology for Adsorption of Chromium from Aqueous Solutions

**DOI:** 10.3390/molecules28114514

**Published:** 2023-06-02

**Authors:** Kongsak Pattarith, David Nugroho, Suwat Nanan, Rachadaporn Benchawattananon

**Affiliations:** 1 Department of Chemistry, Faculty of Science, Buriram Rajabhat University, Buriram 31000, Thailand; 2 Integrated Science, Faculty of Science, Khon Kaen University, Khon Kaen 40002, Thailand; 3 Materials Chemistry Research Center, Department of Chemistry and Center of Excellence for Innovation in Chemistry (PERCH-CIC), Faculty of Science, Khon Kaen University, Khon Kaen 40002, Thailand

**Keywords:** polyethylenimine (PEI), cellulose modification, microwave methodology, biocompatible, adsorption of chromium

## Abstract

A large amount of agricultural waste was used to prepare cellulose (Cel) and then the surface was modified with PEI (Cel-PEI) using the microwave method. To be used as a metal adsorbent, the adsorption of Cr (VI) from an aqueous solution by Cel-PEI was measured using Fourier transform infrared (FTIR) spectroscopy, scanning electron microscopy (SEM), X-ray diffraction (XRD), and thermogravimetric analysis (TGA) techniques. The parameters of Cr (VI) adsorption in solution by the Cel-PEI adsorbent were as follows: the pH of the solution was 3, the concentration of the chromium solution was 100 mg/L, and the adsorption time was 180 min at 30 °C using 0.01 g of adsorbent. Cel-PEI had a Cr (VI) adsorption capacity of 106.60 mg/g, while the unadjusted Cel was 23.40 mg/g and the material recovery showed a decrease in efficiency of 22.19% and 54.27% in the second and third cycles, respectively. The absorption isotherm of chromium adsorption was also observed. The Cel-PEI material conformed to the Langmuir model with an R^2^ value of 0.9997. The kinetics of chromium adsorption showed that under pseudo-second-order analysis, with R^2^ values of 0.9909 and 0.9958 for Cel and Cel-PEI materials, respectively. The G° and H° values of the adsorption process were negative, indicating that the adsorption is spontaneous and that the adsorption process is exothermic. The efficient preparation adsorbent materials for Cr (VI) was achieved using a short microwave method that is low-cost and environmentally friendly for use in the treatment of Cr-contaminated wastewater.

## 1. Introduction

In recent years, environmental pollution has become a serious problem for many countries, as the impact on the population has intensified significantly. The increase in pollution is due to the increase in industrial and agricultural production due to the increasing demands of the population [1,2]. The waste that is released include both organic and inorganic substances. Heavy metals are highly toxic wastes that accumulate in the environment and living organisms and can cause fatal diseases in humans [3,4]. Chromium (VI) is a heavy metal that is classified as very dangerous. It is a hazardous waste from various industries such as leather dyeing, metal plating, production of batteries, paint, and pesticides, etc. Chromium (VI) can be removed using several methods such as chemical precipitation, reverse osmosis biological therapy, coagulation processes, and flocculation [5,6,7,8,9,10,11]. Adsorption is one of the most widely used methods for chromium (VI) removal. It is a simple, inexpensive, and effective method for low metal concentrations [12]. As a result, new sorbent preparations for metal removal were studied. The main purpose was to increase the adsorption efficiency of adsorbent materials, i.e., to increase their ability to remove heavy metals [13,14,15,16].

Cellulose is an agricultural by-product. A large amount is left over each year. Cellulose is a polysaccharide containing hydroxyl groups, which are highly effective functional groups, making it widely used in environmental pollution elimination [17,18,19] in the form of absorbent materials. However, cellulose has a low adsorption capacity, low specificity, and limited reusability [20]. To reduce these limitations and increase the adsorption efficiency of cellulose, the surface can be improved by chemical methods [21,22]. Polyethyleneimine (PEI) is a chemical used to improve absorbent materials. PEI is a water-soluble cationic polyelectrolyte. Additionally, there are many amine groups (N) in its molecular chain. PEI is biocompatible and does not pollute the environment [23]. Because PEI’s amine group is easily protonated in acidic environments, it can adsorb metals by coordination or electrostatic interactions [24].

In recent years, a large amount of research has been conducted using microwaves to prepare various materials, including absorbent material [25,26,27]. The use of microwaves is an efficient process in the preparation of various materials, including absorbent materials, with many advantages since they can be heated quickly and uniformly. Heat is generated throughout the mass of the material, causing the reaction to be rapid, helping to shorten the synthesis time [28,29]. The reaction mixture can be made in an aqueous medium because water molecules easily absorb microwaves [30].

In this research, cellulose fibers were prepared from agricultural waste bamboo and were modified with polyethyleneimine (PEI) using a microwave method. To be used as absorbent material to remove chromium (VI) from wastewater, we studied the suitable conditions and factors including the kinetics and thermodynamics of adsorption. The sorbents were highly effective in removing chromium (VI) from the solution in a short period of time. This process does not use organic solvents, and is cheap and environmentally friendly.

## 2. Results and Discussion

### 2.1. Characterization of Adsorbents

#### 2.1.1. FTIR Analysis

**Figure 1:**
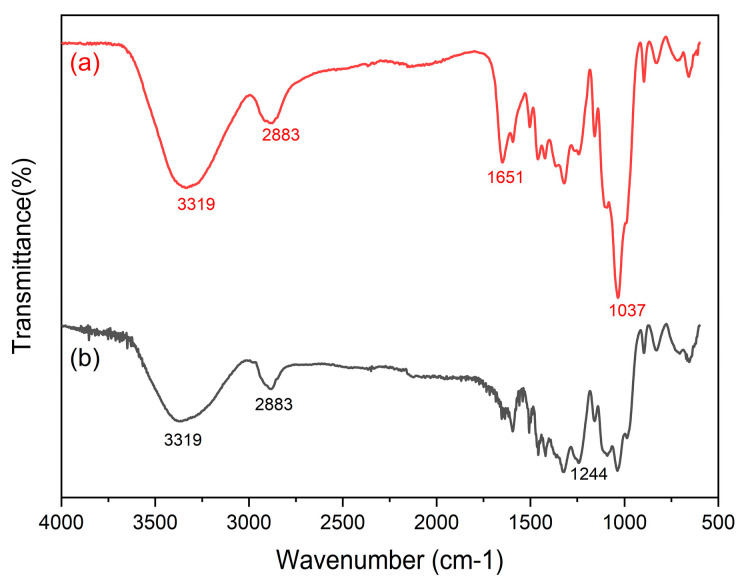
FTIR spectra of Cel and Cel-PEI.

The functional group analysis of the Cel and Cel-PET adsorbents using Fourier transform infrared (FT-IR) spectrometry is shown in Figure 1. Both materials have the same primary functional groups. In Figure 1, the peak at 3319 cm^−1^ is caused by −OH group stretching vibration and the peak at 2883 cm^−1^ is caused by asymmetric C-H stretching [31,32]. The strong peak at 1244 cm^–1^, which was assigned to the C–O–C stretching vibration of the acetyl group, disappeared in the spectrum of cellulose (Figure 1b) [33] while in the FTIR spectra of Cel-PEI (Figure 1b), additional peaks appeared at 1651 cm^−1^ caused by the C≡N stretching vibration, and at 1037 cm^−1^ caused by the C–C skeleton vibration in the PEI-modified cellulose [34].

#### 2.1.2. SEM Analysis

**Figure 2:**
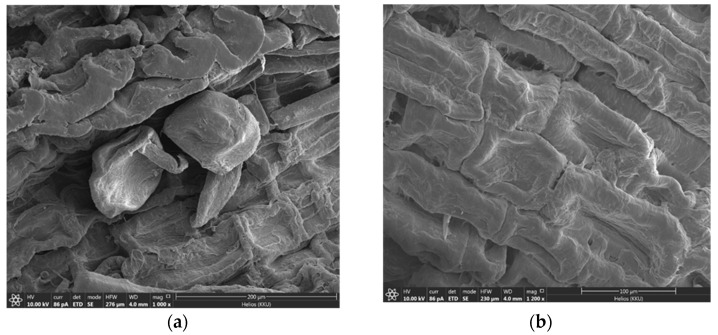
Morphological structure of adsorbents (**a**) cellulose and (**b**) cellulose-PEI.

The scanning electron microscopy (SEM) photographs of the surface morphology of the adsorbents are shown in Figure 2. In Figure 2a, as an example, Cel exhibited cellulose surface characteristics, such as plant cellular characteristics. When the cellulose was treated with PEI (Figure 2b), it can be clearly seen that the PEI film was coated on the surface of the cellulose. This confirmed that the cellulose was surface treated with PEI.

#### 2.1.3. Thermogravimetric and XRD Analyses

**Figure 3:**
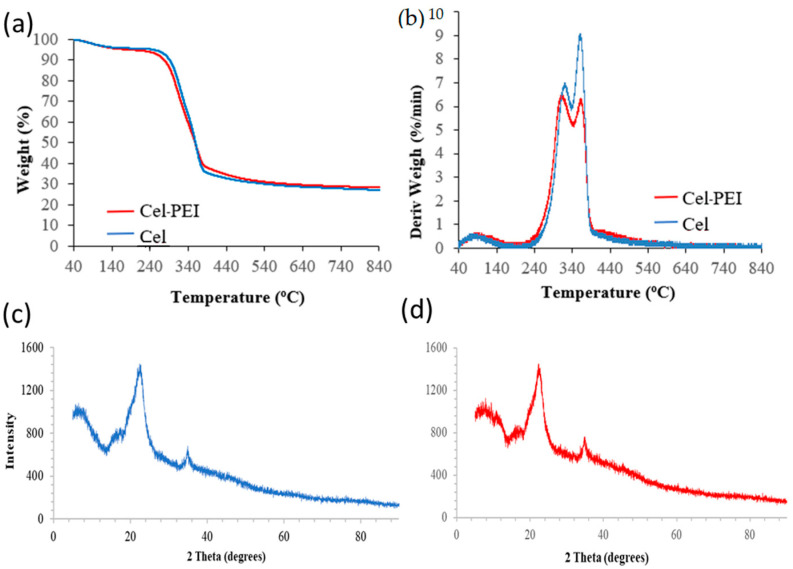
(**a**) Thermogravimetric analysis (TGA) and (**b**) derivative thermogravimetric (DTG) curves of Cel and Cel-PEI. (**c**) XRD of Cel and (**d**) XRD of Cel-PEI.

The thermogravimetric analysis results of the Cel and Cel-PEI sorbents are shown in Figure 3. In Figure 3a,b, the thermogravimetric (TG) and derivative thermogravimetric (DTG) results of Cel and Cel-PEI show that the Cel-PEI materials exhibit zero weight loss by thermal decomposition at slightly lower temperatures compared to Cel, due to the decomposition effect of the PEI used to improve the cellulose. The results of the X-ray diffraction analysis of Cel and Cel-PEI (Figure 3c,d) show that the X-ray diffraction patterns of the two absorbents were similar in terms of band formation. The amplitude at 2θ = 22.25° and 35° which are correlated with cellulose peaks from a previous study [35].

### 2.2. Adsorption Studies

#### 2.2.1. Effect of Solution pH and Time

**Figure 4:**
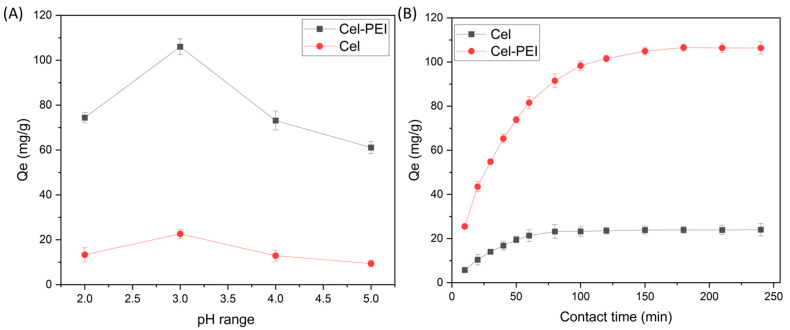
(**A**) The relationship between pH values in the adsorption of Cr (VI) in solution. (**B**) Graph showing the relationship between contact time and the adsorption of Cr (VI) in solution Cel-Cr and Cel-PEI-Cr.

The effect of the pH of the K_2_Cr_2_O_7_ solution on the adsorption capacity of Cel and Cel-PEI was studied in the range of 2–5. The experimental results are shown in Figure 4A. It was found that both Cel and Cel-PEI had the highest Cr (VI) adsorption values at pH 3 with adsorption values of 22.50 and 106.60 mg/g, respectively. Most of the chromium ions were in the form of HCrO_4_^−^ [36] when the pH of the solution is low. When the acidity of the solution increases so that the surface of the adsorbent is protonated with H^+^, the amount of Cr^6+^ binding to the surface is reduced. In the solutions with a higher pH, the chromium ion will be in the form CrO_4_^−^ [36]. The amount of adsorption is reduced because the chromium is less soluble and when the pH is raised further, the chromium will precipitated. The adsorption time and Cr (VI) adsorption capacity of the Cel and Cel-PEI adsorption materials were studied. The adsorption of a constant concentration of chromium is shown in Figure 4B. Using a Cr (VI) concentration of 100 mg/L, it was found that when the adsorption time increased, the metal adsorption of each material was increased until it reached the equilibrium point. The Cr (VI) adsorption equilibrium times of the Cel and Cel-PEI sorbents were 120 and 180 min, respectively.

#### 2.2.2. Effect of Initial Concentration of Cr and Adsorbent Dosage

**Figure 5:**
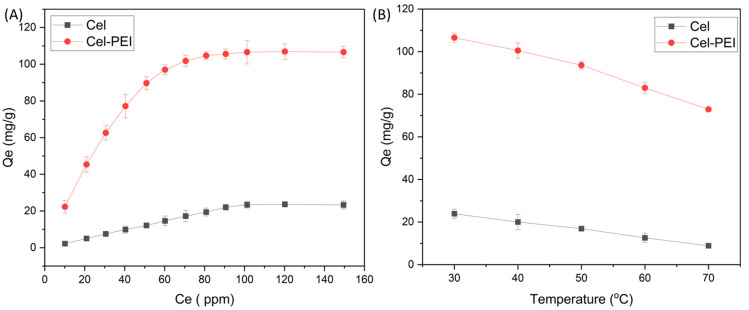
(**A**) The relationship between the initial concentration of K_2_Cr_2_O_7_ and the adsorption of Cr (VI) in solution. (**B**) Graph showing the relationship between temperature and the adsorption of Cr (VI) in solution.

The study of the Cr (VI) adsorption capacity of the Cel and Cel-PEI adsorbents was performed using different concentrations of chromium ions (10–150 mg/L) with 0.010 g of the sorbent. At high concentrations, the diffusion of metal ions in the solution to the surface of the adsorbent occurs rapidly [37]. The maximum Cr (VI) adsorption capacity of the Cel and Cel-PEI materials were 23.50 and 106.60 mg/g, respectively, for a 100 mg/L chromium ion solution, increasing the concentration higher than this did not increase the amount of adsorption. The effect of sorbent content on Cr (VI) adsorption was conducted using an initial concentration of chromium solution of 100 mg/g at pH 3, an adsorption time of 180 min, and temperature of 30 °C. The effect of varying the sorbent amount (0.05, 0.01, and 0.015 g) is shown in Figure 5. When increasing the amount of the Cel and Cel-PEI sorbents, the adsorption capacity increased. This is because the adsorption surface is increased and the dispersion of the adsorbent to the surface is greater [38]. The Cel-PEI adsorbent absorbed 4 times more Cr than Cel when using 0.05 g of adsorbent.

#### 2.2.3. The Efficiency of Adsorbent Recovery

**Figure 6:**
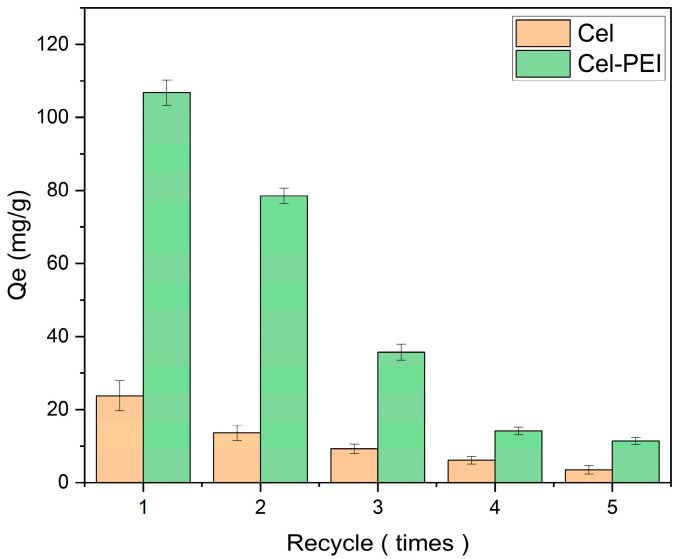
The relationship between the number of times of reuse and the amount of adsorption of Cr (VI) in solution.

The chromium adsorption efficiency of Cel and Cel-PEI in a 100 mg/L K_2_Cr_2_O_7_ solution with an adsorption time of 180 min at 30 °C and using 0.0100 g of adsorbent was studied (Figure 6). Cel-PEI was the most effective in the first cycle of adsorption and decreased in the next cycle. For Cel-PEI, it was found that the efficiency declines in cycle 2 and 3 and was 22.19% and 54.27%, respectively. This may be because some of the PEI used in the surface treatment had come off in the washing process in order to reuse the sorbent. Another reason may be that some of the Cr ions that are tightly bound to the N groups of PEI did not escape during the washing process. Both of these scenarios would cause the efficiency of adsorption to decrease. However, the Cr adsorption efficiency of the Cel-PEI adsorbent was still higher than that of the non-PEI-modified material in all cycles of use.

#### 2.2.4. Absorption Isotherm Studies

The adsorption isotherm shows the relationship of the amount of chromium adsorption and the concentration of chromium solution at equilibrium according to the Langmuir isotherm adsorption model. The isotherms can suggest that the adsorption occurs as a monolayer, the surface of the adsorbent is homogenous and the Freundlich isotherm indicates a multilayer adsorption. The equation for the absorption isotherm is as follows [39,40]:

Langmuir isotherm equation:

(1) $$\frac{C_{e}}{Q_{e}}=\frac{1}{{\mathrm{bQ}}_{m}}+\frac{C_{e}}{Q_{m}}$$

Freundlich isotherm equation:

(2) $${\mathrm{lnQ}}_{e}={\mathrm{lnK}}_{f}+\frac{1}{n}{\mathrm{lnC}}_{e}$$

where C_e_ (ppm) is the equilibrium concentration of the heavy metals in the liquid phase, Q_e_ (mg/g) is the solid phase equilibrium concentration of heavy metals, Q_m_ is the Langmuir monolayer adsorption capacity, b (L/mg) is the Langmuir equilibrium adsorption constant, and K_f_ and n are the Freundlich equilibrium adsorption constants.

**Figure 7:**
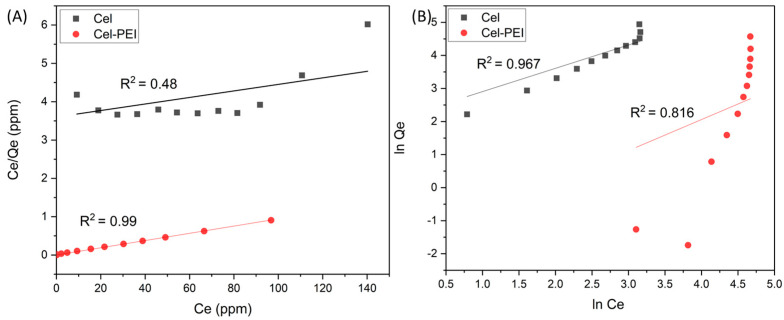
Langmuir (**A**) and Freundlich (**B**) isotherm plots for Cr (VI) adsorption onto Cel and Cel-PEI.

The experimental results are shown in Figure 7 and the parameters are presented in Table 1. When comparing the correlation coefficients of the two materials, the Cr (VI) adsorption mechanism of the Cel material followed the Freundlich model. The correlation coefficient was 0.9671, which indicates the unevenness of the material surface. For Cel-PEI, the adsorption mechanism corresponds to the Langmuir model with a correlation coefficient of 0.9997, indicating that the PEI-modified surface is homogeneous and has a monolayer adsorption mechanism [41].

#### 2.2.5. Kinetics of Chromium Adsorption

The kinetics of adsorption was studied by fitted the data to those from kinetic experiments according to the following equations [42,43]:

Pseudo-first-order:

(3) $$\ln(Q_{e}-Q_{t})={\mathrm{lnQ}}_{e}-(k_{1})t$$

Pseudo-second-order:

(4) $$\frac{t}{Q_{t}}=\frac{1}{k_{2}Q_{e}^{2}}+\frac{t}{Q_{e}}$$

where Q_e_ and Q_t_ are the amount of adsorbed in mg/g at equilibrium and at time t (min), respectively, k_1_ (min^−1^) is the rate constant of the pseudo-first-order adsorption, and k_2_ (g mg^−1^ min^−1^) is the pseudo-second-order rate constant.

**Figure 8:**
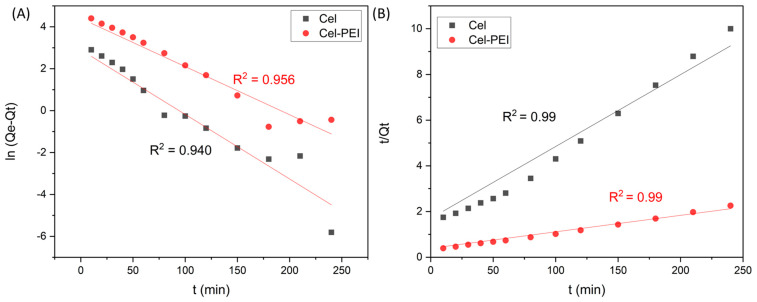
Pseudo-first-order kinetic (**A**) and pseudo-second-order kinetic (**B**) models for adsorption of Cr (VI) onto Cel and Cel-PEI.

The study of the adsorption kinetics using the two equations is shown in Figure 8 and the various constants are shown in Table 2. The adsorption kinetics of the Cel and Cel-PEI materials complied with the pseudo-second-order model with correlation coefficients of 0.9909 and 0.9958, respectively, which are higher compared to the correlation coefficient of the pseudo-first-order model.

#### 2.2.6. Thermodynamic Studies

The thermodynamic parameter values for adsorption are ΔG° (standard free energy), ΔH° (enthalpy change), and ΔS° (entropy change), which can indicate the change in heat occurring in the adsorption process. These values can be obtained from the slope and the intercept of the graph plotted between the ln KC value and the 1/T value according to the van’t Hoff equation [44,45,46].

(5) $$\ln\mathrm{Kc}=\frac{\Delta H{}^{\circ}}{\mathrm{RT}}+\frac{\Delta S{}^{\circ}}{R}$$

(6) $$K_{c}=\;\frac{q_{e}}{C_{e}}$$

The ΔG° can be calculated using the follow equation [46]:

ΔG° = ΔH° − TΔS° (7)

where R^2^ is the gas constant (8.314 J mol^−1^ K^−1^) and T is the absolute temperature (K).

**Figure 9:**
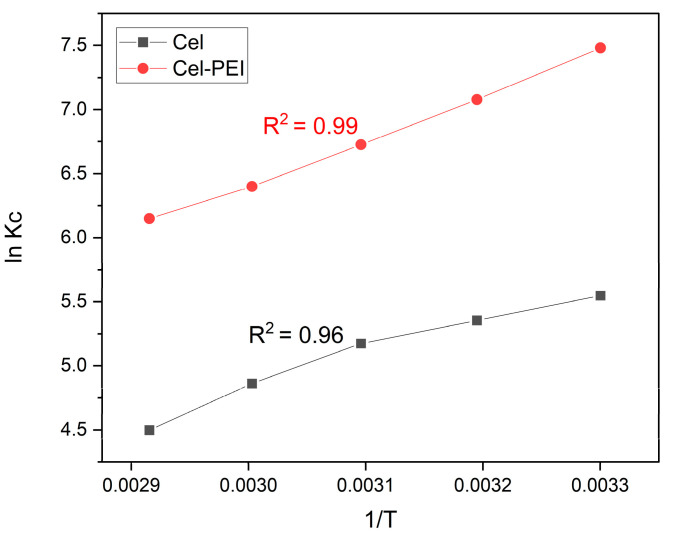
Van’t Hoff plot for adsorption of Cr (VI) on Cel and Cel-PEI.

Figure 9 shows the experimental results according to the van’t Hoff equation to calculate the various thermodynamic values (Table 3); it was found that ΔG° and ΔH° were negative values, indicating that the Cr (VI) adsorption process on the material was exothermic and spontaneous. The results also show that the temperature increased, indicating that the adsorption process is poor.

## 3. Materials and Methods

### 3.1. Materials and Characterization

Polyethyleneimine (PEI), sodium hydroxide (NaOH), sodium hypochlorite (NaClO), and a chromium standard solution (1000 mg L^−1^) were purchased from Merck Co., Inc. (Darmstadt, Germany). The cellulose fibers (Cel) were derived from bamboo waste. All chemicals were of analytical grade and were used without purification.

A Thermo Scientific spectrometer (Thermo Nexus 470FT-IR, Nicorette, Waltham, MA, USA) was used to obtain the Fourier transform infrared (FT-IR) spectra of the different materials in the range 400–4000 cm^−1^, while a scanning electron microscope (Hitachi FE-SEMS-4800, Hitachi City, Japan) was used to determine their surface morphologies. The phase purity and crystal structure were examined using an X-ray diffractometer (Bruker/D8 Advance, Billerica, MA, USA). Thermogravimetric analyses (TGA) were performed using a Thermo Scientific SDT Q600 series Thermogravimetric Analyzer (TA instrument, New Castle, DL, USA). The concentration of chromium was determined using an atomic absorption spectrophotometer (PerkinElmer PinAAcle 900, Waltham, MA, USA).

### 3.2. Preparation of Cellulose Fibers

The bamboo waste was soaked in a 30% sodium hydroxide solution for 1 week and then the sodium hydroxide solution was completely washed off. The fibers were boiled in a 5% sodium hypochlorite solution for 1 h to remove lignin and hemicellulose. The fibers were washed with DI water, dried at 60 °C for 24 h, and spun with a blender to obtain cellulose fibers (Cel) which were stored in a desiccant jar until use in experiments.

#### Cellulose Fiber Improvement Using Microwave Method

In a round bottom flask, 1 g of cellulose fiber was placed, 125 mL of water was added, the fibers were disperse using an ultrasonicator, 5 g of polyethyleneimine (PEI) was added, and the mixture was refluxed. The mixture was heated using microwave waves (800 W) for 10 min, after which it was washed with DI water and dried at 60 °C for 24 h to obtain PEI-modified cellulose fiber absorbent (Cel-PEI).

### 3.3. Study of Factors Affecting the Batch Adsorption of Chromium by the Sorbent Material

#### 3.3.1. pH Value

The effect of the pH of the metal solution on adsorption was studied using 0.010 g of adsorbent in a 50 mL solution with an initial concentration of 250 mg/L chromium. The solution was adjusted to different pH values (pH 2–5) and then placed in a shaker at 150 rpm and the temperature was controlled at 30 °C for 180 min. After that, the solution was centrifuged. The clear solution was collected and analyzed for chromium content in order to calculate the adsorption amount. 

#### 3.3.2. Adsorption Time

The adsorption time is the time it takes the adsorption process to reach an equilibrium. This was studied using 0.010 g of adsorbent in a 50 mL solution with an initial concentration of 100 mg/L chromium. The solution was adjusted to a pH of 3 and was placed in a shaker at 150 rpm and the temperature was controlled at 30 °C for 180 min. After that, the solution was centrifuged. The clear solution was collected and analyzed for chromium content in order to calculate the adsorption amount.

#### 3.3.3. Initial Concentration Affecting Adsorption

The concentration of chromium in the solution is an important factor for the adsorption amount. This was studied by taking 0.010 g of adsorbent in 50 mL solutions with different initial concentrations of chromium (10–150 mg/L). The solution was adjusted to a pH of 3 and was placed in a shaker at 150 rpm and the temperature was controlled at 30 °C for 180 min. 

#### 3.3.4. Affecting Adsorption Temperature

The adsorption temperature was studied using 0.010 g of sorbent in a 50 mL solution with an initial concentration of 100 mg/L chromium, adjusted to a pH of 3. Then, with the solution was placed in a shaker at 150 rpm and the temperature was controlled at different temperatures of 30, 40, 50, and 60 °C for 180 min. After that, the solution was centrifuged and analyzed for chromium content to calculate the amount of adsorption.

#### 3.3.5. Amount of Absorbent Material

Different amounts of adsorbent were used (0.0050, 0.010, and 0.015 g) to adsorb chromium in a 50 mL solution with an initial concentration of 100 mg/L chromium. The solution was adjusted to a pH of 3, and then placed in a shaker at 150 rpm and at 30 °C for 180 min. The clear solution was centrifuged to analyze the amount of chromium in order to calculate the amount of adsorption.

### 3.4. Reuse of Absorbent Material

The sorbent recycling study was performed using 0.010 g of sorbent in a 50 mL solution with an initial concentration of 100 mg/L chromium, adjusted to a pH of 3, and placed in a shaker at 150 rpm at 30 °C for 180 min. After that, the clear solution was centrifuged to analyze the chromium content to calculate the adsorption. The used absorbent material was placed into 100 mL of 0.1 M hydrochloric acid and incubated at room temperature for 12 h. Afterwards, the material was washed with DI water until the pH was 7. The washed adsorbent was dried at 60 °C for 12 h. The absorbent materials were reused 5 times.

### 3.5. Analysis of Chromium Adsorption

The amount of chromium adsorption by the sorbent can be calculated from the decrease in the concentration of the initial metal solution after adsorption with respect to the weight of the sorbent used according to the equation [31]:

(8) $$Q_{e}=\frac{(C0\;-\;\mathrm{Ce})\;V}{m}$$

where Q_e_ represents the adsorption capacity, C_0_ and C_e_ represent the initial and equilibrium concentrations of Cr (VI), respectively, V represents the volume of the solution, and m represents the weight of the adsorbent used (0.05 g).

## 4. Conclusions

A Cel-PEI absorbent material was obtained by improving cellulose with PEI using a microwave technique. To be used as Cr (VI) adsorbent, the characteristics of the adsorbent were studied using various techniques such as FTIR, SEM, TGA, XPS, and XRD. The optimal conditions for Cel-PEI adsorption were a pH of 3, chromium solution concentration of 100 mg/L, and adsorption time of 180 min at 30 °C. Using 0.01 g of adsorbent, Cel-PEI had an adsorption capacity of 106.60 mg/g which is higher than that of the unmodified Cel material (23.40 mg/g). The study of the reuse of the material found that the efficiency decreased by 22.19% and 54.27% in the 2nd and 3rd cycles of adsorption, respectively. The absorption isotherm effect of chromium adsorption by the Cel-PEI material according to Langmuir’s model had an R^2^ value of 0.9997, which indicates that the process is a monolayer adsorption and the adsorbent surface is homogeneous. The kinetics of chromium adsorption results were studied; it is a pseudo-second-order reaction, with R^2^ values of 0.9909 and 0.9958 for Cel and Cel-PEI materials, respectively. These results indicate that the adsorption rates depend on both the concentration of the chromium solution and the amount of adsorbent. The ΔG° and ΔH° values were calculated by plotting the equations. The van’t Hoff value was negative, indicating that the adsorption is spontaneous and that the adsorption process is exothermic, with decreased adsorption efficiency under high temperature conditions.

## Figures and Tables

**Table 1:** Constants and correlation coefficients of Langmuir isotherm and Freundlich isotherm models.

Adsorbent	Langmuir			Freundlich		
	Q_m_ (mg/g)	b (mL/mg)	R^2^	K_f_ (L/g)	n (L/mg)	R^2^
Cel	81.3008	0.0038	0.4868	2.8471	0.9557	0.9671
Cel-PEI	108.6957	0.7480	0.9997	49.6352	0.2561	0.8168

**Table 2:** Parameters of the pseudo-first and pseudo-second-order reaction equations.

Absorbent	Pseudo-First-Order Model			Pseudo-Second-Order Model		
	k_1_ (min^−1^)	Q_e_, cal (mg/g)	R^2^	k_2_ (g/mg min)	Q_e_, cal (mg/g)	R^2^
Cel	0.0322	16.28	0.9402	0.0019	33.67	0.9909
Cel-PEI	0.0244	74.03	0.9596	0.0003	138.88	0.9958

**Table 3:** Thermodynamic parameter of adsorption of Cr (VI) on Cel and Cel-PEI.

	$\Delta G{}^{\circ}\;\;(\mathrm{KJ}\;{\mathrm{mol}}^{-1})$				ln K_c_				ΔH°	ΔS°
	30 °C	40 °C	50 °C	60 °C	30 °C	40 °C	50 °C	60 °C	(KJ mol^−1^)	(KJ mol^−1^)
Cel	−13.97	−13.93	−13.89	−13.45	5.54	5.35	5.17	4.86	−22.26	−0.02
Cel-PEI	−19.46	−18.41	−18.06	−17.71	7.48	7.07	6.72	6.39	−36.42	−0.03

## Data Availability

The data are available upon request from the authors.

## Sample Availability

Samples are available from the authors.
